# Development of titanium dioxide nanowire incorporated poly(vinylidene fluoride–trifluoroethylene) scaffolds for bone tissue engineering applications

**DOI:** 10.1007/s10856-019-6300-4

**Published:** 2019-08-14

**Authors:** Anitha Augustine, Robin Augustine, Anwarul Hasan, Varun Raghuveeran, Didier Rouxel, Nandakumar Kalarikkal, Sabu Thomas

**Affiliations:** 1 International and Inter University Centre for Nanoscience and Nanotechnology, Mahatma Gandhi University, Kottayam, Kerala 686 560 India; 2 Department of Chemistry, Bishop Kurialacherry College for Women, Amalagiri, Kottayam, Kerala 686561 India; 3 Department of Mechanical and Industrial Engineering, College of Engineering, Qatar University, 2713 Doha, Qatar; 4 Biomedical Research Centre, Qatar University, 2713 Doha, Qatar; 5 MIMS Research Foundation, Malabar Institute of Medical Sciences (Aster MIMS), Kozhikode, Kerala 673016 India; 6 Université de Lorraine, CNRS, IJL, F-54000 Nancy, France; 7 School of Pure and Applied Physics, Mahatma Gandhi University, Kottayam, Kerala 686 560 India; 8 School of Chemical Sciences, Mahatma Gandhi University, Kottayam, Kerala 686 560 India

## Abstract

Critical size bone defects that do not heal spontaneously are among the major reasons for the disability in majority of people with locomotor disabilities. Tissue engineering has become a promising approach for repairing such large tissue injuries including critical size bone defects. Three-dimension (3D) porous scaffolds based on piezoelectric polymers like poly(vinylidene fluoride-trifluoroethylene) (P(VDF-TrFE)) have received a lot of attention in bone tissue engineering due to their favorable osteogenic properties. Owing to the favourable redox properties, titanium dioxide (TiO_2_) nanostructures have gained a great deal of attention in bone tissue engineering. In this paper, tissue engineering scaffolds based on P(VDF-TrFE) loaded with TiO_2_ nanowires (TNW) were developed and evaluated for bone tissue engineering. Wet-chemical method was used for the synthesis of TNW. Obtained TNW were thoroughly characterized for the physicochemical and morphological properties using techniques such as X-Ray diffraction (XRD) analysis and transmission electron microscopy (TEM). Electrospinning was used to produce TNW incorporated P(VDF-TrFE) scaffolds. Developed scaffolds were characterized by state of art techniques such as Scanning Electron Microscopy (SEM), XRD and Differential scanning calorimetry (DSC) analyses. TEM analysis revealed that the obtained TiO_2_ nanostructures possess nanofibrous morphology with an average diameter of 26 ± 4 nm. Results of characterization of nanocomposite scaffolds confirmed the effective loading of TNW in P(VDF-TrFE) matrix. Fabricated P(VDF-TrFE)/TNW scaffolds possessed good mechanical strength and cytocompatibility. Osteoblast like cells showed higher adhesion and proliferation on the nanocomposite scaffolds. This investigation revealed that the developed P(VDF-TrFE) scaffolds containing TNW can be used as potential scaffolds for bone tissue engineering applications.

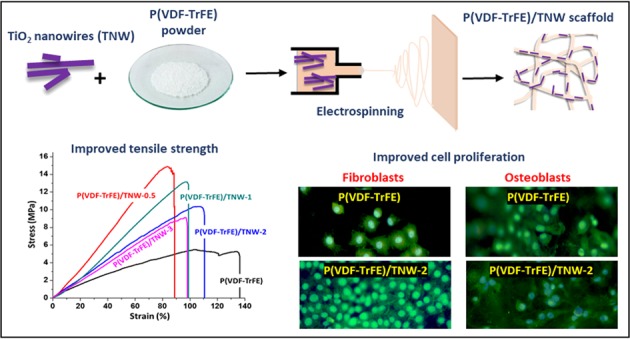

## Introduction

Accidental trauma, sports injuries, developmental deformities, tumor resection and infection can lead to significant loss of bone tissue which cannot be repaired naturally. These critical size bone defects form the major reason for the disability in several people with locomotor disabilities. Bone tissue engineering is emerging as a promising option to regenerate or repair large tissue defects without the potential limitations associated with autologous and allogeneic bone grafts like disease transmission, immune rejections and lack of availability [1, 2]. Ideally, bone tissue engineering aims to regenerate the natural bone which is a porous framework mainly consisting of collagen fibers that run through hydroxyapatite and living cells such as osteoblasts. The main goal of bone tissue engineering is the generation of a functional bone tissue in the defected area by using a combination of polymeric scaffolds, cells, and signalling cues [3, 4].

The use of electrospun polymeric biomaterials for bone tissue engineering applications attained considerable importance owing to their ability to act as porous 3D supports for cell adhesion and proliferation [5, 6]. Poly(vinylidene fluoride-co-trifluoroethylene) copolymer [P(VDF-TrFE)] has huge potential as scaffold for tissue engineering applications due to its piezoelectric property and biocompatibility [7]. In piezoelectric materials such P(VDF-TrFE), even small vibration or mechanical stretching during the muscular movement can produce transient surface charges which can help in cell adhesion and proliferation. Among the four different crystalline phases in P(VDF-TrFE) such as α, β, γ, and δ, electroactive β phase plays the key role in facilitating cell proliferation [8, 9]. Earlier studies demonstrated the potential of P(VDF-TrFE) based scaffolds for the ability to repair or regenerate damaged skeletal muscles and spinal cord defects [10, 11]. Moreover, electrospun P(VDF-TrFE) scaffold along with cardiovascular cells was reported as a suitable biomaterial for the cardiovascular tissue engineering [12]. In addition, several studies showed that the physicomechanical properties of these piezoelectric polymers can be improved or tuned by the incorporation of nanofillers [13, 14].

Various nanoparticles have been used in polymeric biomaterials to improve their biological performance [15–18]. Titanium dioxide (TiO_2_) nanostructures have several applications in many areas such as in dye-sensitized solar cells, photocatalysis, and biomedical equipment [19, 20]. Both in vitro cell culture studies and animal experiments demonstrated that TiO_2_ nanoparticles can encourage cell migration depending upon the effective concentration and the size of nanoparticles [21, 22]. Application of TiO_2_ nanotubes for bone regeneration was extensively studied by Adhikari et al. [23]. TiO_2_ nanostructures can activate macrophages which will trigger cell migration and repair/regeneration of damaged tissue [24]. Since the photocatalytic reactions occurs on the surface of TiO_2_ nanostructures play the main role in the cellular response, a high surface area-to-volume ratio is vital for getting a desired outcome [25]. Unlike the other morphological forms of TiO_2_ (nanopowders, nanotubes and nanowires), TiO_2_ nanowires (TNW) are highly promising due to their high surface area to volume ratio and resulting superior catalytic performance. Studies have shown that the TiO_2_ nanoparticles can induce the generation of harmful reactive oxygen species (ROS) in biological system [26]. Thus, the use of such nanoparticles in biological system should be in a highly controlled manner to avoid the deleterious effects due to the excess ROS generated. However, under normal physiological conditions, ROS participates in several important biomolecular signalling pathways [27]. Although unnecessary and higher production of ROS can result in deleterious effects, an optimal level can exert beneficial outcomes in tissue regeneration and repair [28]. Earlier report suggests that incorporation of TiO_2_ nanoparticles in scaffolds can improve adhesion of osteoblast cells on the scaffolds [29]. Our recent report suggests that incorporation of TiO_2_ nanorods can improve the cell adhesion, proliferation, angiogenic and wound healing potential of electrospun polycaprolactone (PCL) scaffolds [30]. A study by Lin and co-workers have shown that the loading of TiO_2_ nanoparticles in P(VDF-TrFE) copolymer results in a considerable improvement in dielectric properties compared to neat ferroelectric copolymer [31]. Thus, incorporation of TNW in P(VDF-TrFE) scaffolds may result in greater cell adhesion and cell viability which are necessary for the success of an engineered bone construct.

Herein this paper, we describe the design and development of highly porous electrospun piezoelectric tissue engineering scaffolds based on P(VDF-TrFE) and TNW with superior ability to support fibroblast and osteoblast like cell proliferation.

## Experimental

### Materials

P(VDF-TrFE) (70/30 PVDF/TrFE ratio) was supplied by Piezotech SAS, France. TiO_2_ nanoparticles (Average diameter 25 nm), MTT (3-(4,5-dimethylthiazol-2-yl)-2,5-diphenyltetrazolium bromide) and DAPI (4,6-diamidino-2-phenylindole) were purchased from Sigma Aldrich, USA. Phalloidin was obtained from Applied Biosystems, USA. Fetal Bovine Serum (FBS), Antibiotic-Antimycotic solution and Dulbecco’s Modified Eagles Medium (DMEM) were purchased from Himedia, India. Acetone, N, N-Dimethyl formamide (DMF) and Paraformaldehyde was obtained from Merck (India).

### Synthesis of TiO_2_ nanowires

The wet chemical method was used for the synthesis of TNW using commercial TiO_2_ nanoparticle as the TiO_2_ precursor as described earlier with slight modifications [32]. The reaction mixture was composed of NaOH aqueous solution (10 mol/l, 20 ml) and the TiO_2_ precursor (0.2 g). This mixture was kept for hydrothermal reaction at 160 °C for 24 h in a Teflon-coated autoclave and then permitted to slowly cooldown to room temperature. Obtained white precipitate was sequentially washed in water and ethanol until the pH value reached 7. This nanoparticle suspension was centrifuged (8000 rpm) and dried at 60 °C. Calcination was performed at 500 °C for 6 h.

XRD analysis was performed to determine the crystalline nature and the structural characteristics of TNW (see Section 2.4.3 for details). High Resolution Transmission Electron Microscope, HR-TEM (JEOL JEM-2100) was used to assess the size distribution and morphological features of synthesized TiO_2_ nanowires.

### Fabrication of electrospun P(VDF-TrFE)/TNW nanocomposites

Electrospinning technology was used to develop P(VDF-TrFE)/TNW nanocomposite scaffolds. Electrospinning instrument was supplied by Holmarc, India. DMF: acetone solvent mixture was used to prepare P(VDF-TrFE) solutions with varying concentration of TNW. Then, composite scaffolds with 0, 0.5, 1, 2 and 3% w/w of TNW with respect to the polymer hereafter referred as P(VDF-TrFE), P(VDF-TrFE)/TNW-0.5, P(VDF-TrFE)/TNW-1, P(VDF-TrFE)/TNW-2, and P(VDF-TrFE)/TNW-3) respectively were fabricated. 10 ml of the polymer/nanoparticle suspensions were used for making each scaffold. Total polymer or polymer/nanofiller concentration was maintained as 15% w/v. The flow rate of the solution, tip to collector distance and the applied DC voltage were set as 1 ml/h, 15 cm and 15 kV respectively. After the completion of the spinning process, electrospun scaffolds were collected and used for characterizations.

### Characterization of P(VDF-TrFE)/TNW nanocomposite scaffolds

#### Scanning electron microscopy (SEM) analysis

Morphology of developed scaffolds were characterized using scanning electron microscopy (SEM). Samples were coated with gold and analysed using a JEOL JSM 6390 scanning electron microscope at 30 kV. ImageJ software was used to measure the fiber diameter. Fiber diameter distribution and average fiber diameter were determined from the diameters of 100 fibers at random positions.

#### Fourier-transform infrared (FTIR) analysis

Both neat and P(VDF-TrFE)/TNW nanocomposite scaffolds were subjected to FTIR analysis to find out the variation in crystalline phases due to TNW incorporation. Analysis was performed using a Perkin Elmer (USA), spectrum 400 FTIR spectrometer with PIKE Gladi ATR (USA) attachment and DTGS detector (on a diamond crystal). Analysis was performed at a scan rate of 15 scans and a resolution of 4 cm^−1^. Data were collected between 450–3500 cm^−1^ with 15 scans at 4 cm^−1^ resolution.

The β-phase fraction in the scaffolds were determined by Lambert–Beer law as given in Eq. (1).

1 $${\mathrm{F}}\left( {\mathrm{\beta }} \right) = \frac{{{\mathrm{A\beta }}}}{{\left( {\frac{{K{\mathrm{\beta }}}}{{{\mathrm{K}}\alpha }}} \right){\mathrm{A\alpha + A\beta }}}}$$

where, F(β) is the β-phase fraction, Aα is the absorbance at 764 cm^−1^ and Aβ is the absorbance at 840 cm^−1^. Kα (6.1 × 10^4^ cm^2^ mol^−1^) and Kβ (7.7 × 10^4^ cm^2^ mol^−1^) are the absorption coefficients at 764 and 840 cm^−1^ respectively.

#### X-ray diffraction (XRD) analysis

XRD analysis of P(VDF-TrFE) and nanocomposite scaffolds were performed using PANalyticalX’Pert Pro X-Ray diffractometer. The current and applied voltage were 30 mA and 45 kV respectively.

#### Differential scanning calorimetry (DSC)

DSC analysis was carried out in two steps using a Perkin Elmer, Diamond DSC apparatus. In the first step, scaffold samples (5 mg) were heated from −50 to +250 °C at 10 °C/min in the presence of nitrogen flow (20 ml/min). In order to remove the thermal history, samples were kept at +250 °C for 1 min and then cooled at the rate of 10 °C/min to −50 °C. In the second step, samples were again heated to +250 °C and cooled to −50 °C under above mentioned conditions. From the heating ramp, thermal properties such as melting temperature (T_m_) and ferroelectric-paraelectric transition temperature (T_F-P_) were obtained. From the cooling ramp, the crystallization temperature (T_c_) and the paraelectric-ferroelectric transition temperature (T_P-F_) were calculated.

#### Mechanical strength and strain characteristics

Uniaxial tensile testing was used to find out the mechanical strength and strain characteristics of the scaffolds. Tensile characteristics of the samples such as maximum elongation, ultimate tensile strength and Young’s modulus (MPa) were determined from the stress-strain curves. Tests were carried out using a Tinus Olsen H50 KT Universal Testing Machine by adhering to ASTM D 882 standard (500 N load cell, 1 mm/min crosshead speed) on rectangular samples (6 × 1 cm size, 1 ± 0.4 mm thickness). Young’s moduli were calculated from the slope of the linear region of the stress-strain curves. Average values of tensile properties were calculated from five tests.

#### In vitro cell adhesion and cell viability studies

UMR-106 rat osteoblast like cells and mouse L-929 fibroblast cells were used to evaluate the cell adhesion and proliferation on neat and nanocomposite scaffolds. Both the cell lines were supplied by National Centre for Cell Science (NCCS), Pune, India. The scaffolds were cut into 1 × 1 cm size and sterilized by 70% ethanol treatment for 20 min and subsequent UV exposure for 20 min. The scaffolds were prewetted in DMEM medium overnight. Osteoblast like cells (50,000 cells/cm^2^) and fibroblasts were seeded on the scaffolds and cultured in 24 well plates with 0.5 ml suitable media (DMEM containing 10% FBS and 1% Antibiotic-Antimycotic solution) for 24 h at 37 °C with 5% CO_2_ supply. Cell seeded control wells without any samples were also maintained. To observe the cell adhesion, samples were fixed with 4% paraformaldehyde, stained with 4,6-diamidino-2-phenylindole (DAPI) and phalloidin. A fluorescent microscope (Leica DMI 3000B, Germany) was used to capture the images. To determine the viability of the cells grown on the scaffolds, MTT cell viability assay was performed after 24 h, 3 days and 7 days based on the manufacturers protocol (n = 3).

### Statistical analysis

In order to find out the statistical significance of hypotheses, un-paired Student’s *t*-test and “One-way ANOVA” were carried out using GraphPad- Prism. A P value less than 0.05 considered as a determinant of significant difference between tested groups.

## Results

### Characterization of TNW

**Fig. 1:**
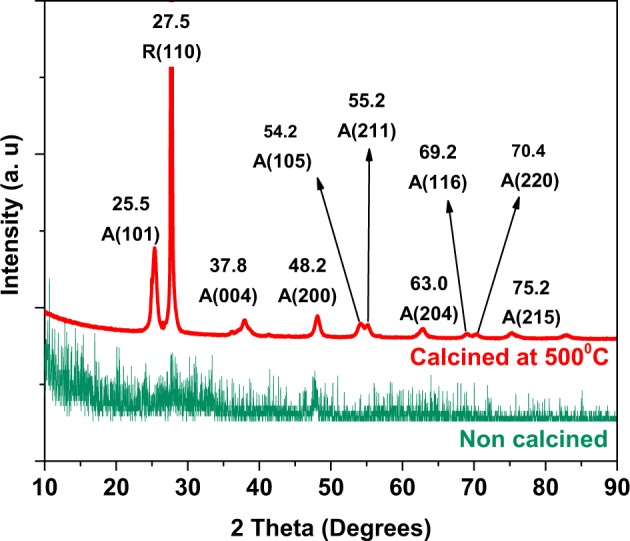
XRD pattern of TNW which were calcined at 500 °C. Parentheses ‘A’ and ‘R’ denotes anatase and rutile crystalline phases, respectively

XRD pattern (Fig. 1) of TNW after calcination at 500 °C for 6 h shows major diffraction patterns located at 2θ = 25.5°, 37.8°, 48.2°, 54.2°, 55.2°, 63.0°, 69.2°, 70.4° and 75.2 corresponding to (101), (004), (200), (105), (211), (204), (116), (220) and (215) planes corresponds to anatase phase of TiO_2_, respectively. Other peak located at 2θ = 27.58° was due to (110) plane of rutile phase [33]. Other peaks of rutile phase were not clearly visible in the XRD pattern. Non calcined samples showed only a few peaks which were undistinguishable from the background scattering, suggesting the amorphous structure.

**Fig. 2:**
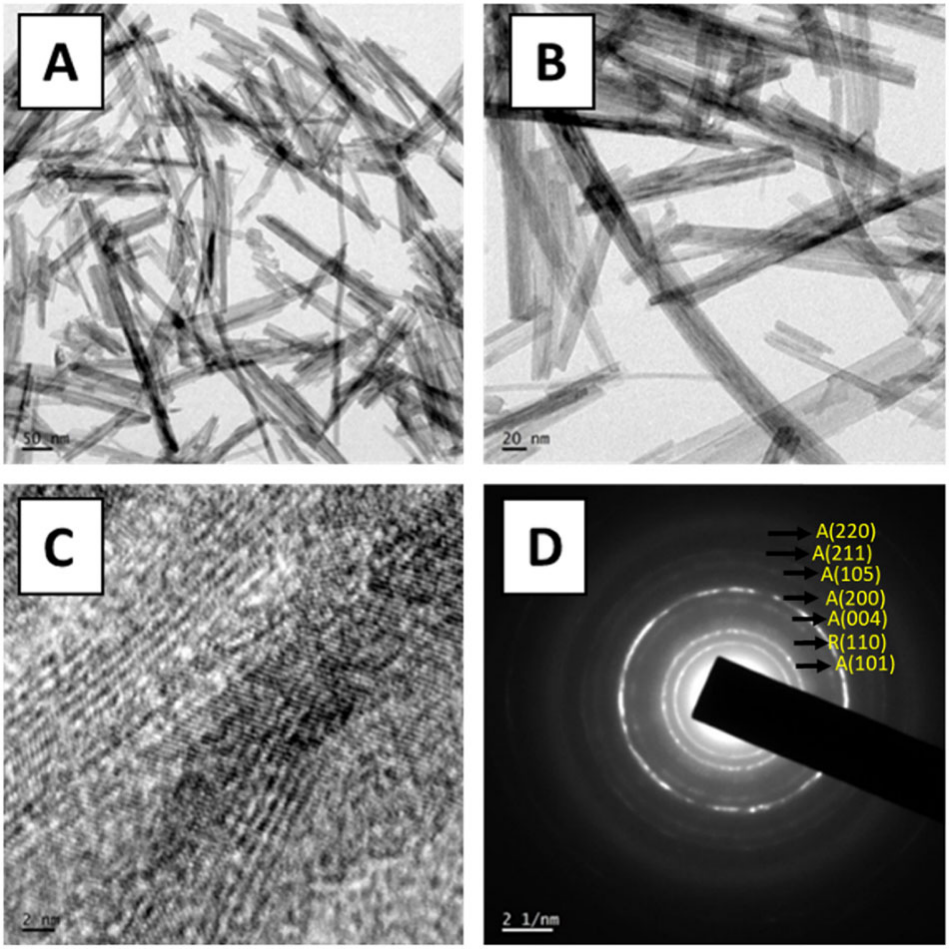
Morphological and structural features of the synthesized materials. Representative TEM images at two different magnifications (**a**, **b**), HR-TEM image (**c**) and SAED pattern (**d**) of TNW. Parentheses ‘A’ and ‘R’ in the labels of (d) denotes anatase and rutile crystalline phases, respectively

TEM images provided the details such as morphology and size distribution of the synthesized TNW. The TEM image demonstrated that the obtained nanostructures possess fiber like elongated morphological features with mean diameter of 25 ± 5 nm (Fig. 2a). The length of the nanowires varied from 100 nm to several micrometers. Higher magnification image showed that each nanowire was formed by the merger of many small fibers with average diameter of 2.4 ± 1.2 nm (Fig. 2b). Interestingly, each such small fiber unit were formed by the union of several monocrystalline units (Fig. 2c). Obtained SAED result shows the diffraction pattern of TiO_2_ nanostructures that mostly composed of anatase phase [34] (Fig. 2d). However, the presence of (110) plane indicate the coexistence of rutile phase also.

### Basic characterization of electrospun P(VDF-TrFE)/TNW membranes

#### Morphology of scaffolds

**Fig. 3:**
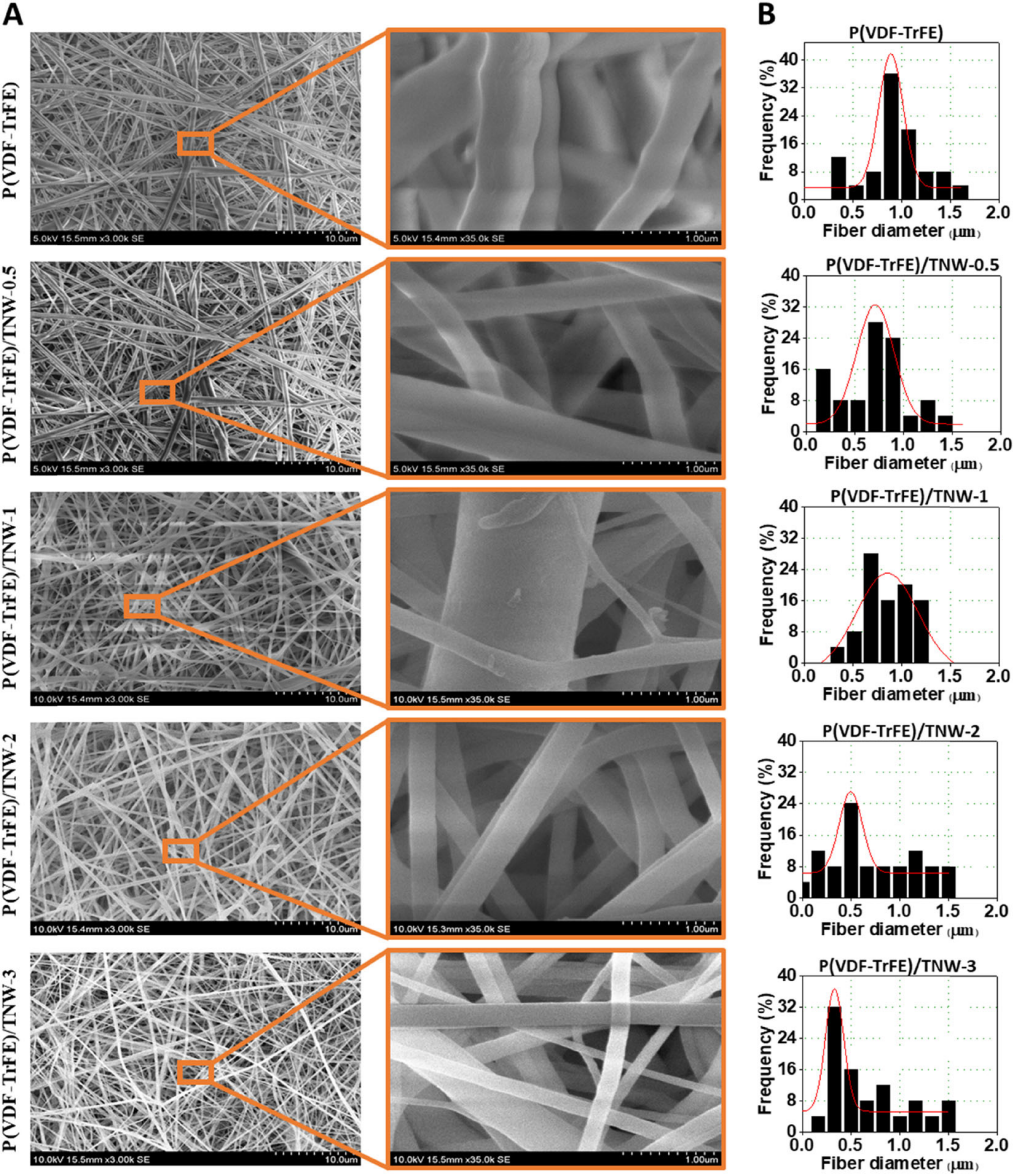
Morphology of developed P(VDF-TrFE) scaffolds containing various amounts of TNW. SEM images (**a**) and fiber diameter distribution graphs (**b**) of P(VDF-TrFE) and P(VDF-TrFE)/TNW nanocomposite scaffolds

Morphological characteristics of the scaffolds are presented in Fig. 3a. The porous scaffolds were composed of randomly oriented fibers with good pore interconnectivity. Individual fiber diameter was measured from SEM micrographs and the fiber diameter distribution graphs are provided in Fig. 3b. Average fiber diameter of electrospun P(VDF-TrFE) scaffolds were slightly reduced with the higher loading of TNW (Table 1).

**Table 1:** Fiber diameter distribution of P(VDF-TrFE) membranes

Sample	Average fiber diameter ± S.D. (nm)
P(VDF-TrFE)	596 ± 201
P(VDF-TrFE)/TNW-0.5	460 ± 104
P(VDF-TrFE)/TNW-1	477 ± 117
P(VDF-TrFE)/TNW-2	437 ± 116
P(VDF-TrFE)/TNW-3	484 ± 112

#### FTIR analysis

**Fig. 4:**
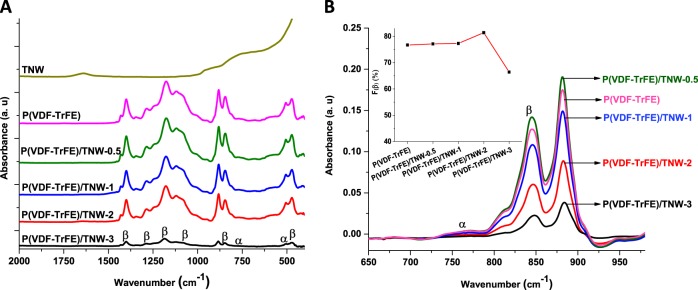
FTIR spectra of bare P(VDF-TrFE) and P(VDF-TrFE)/TNW scaffolds (**a**) and a magnified view of the FTIR bands from 650 to 980 cm^−1^ demonstrating the change in intensity of α and β phase signals upon the incorporation of TNW (**b**). Inset in b shows the F(β) in scaffold with various TNW loadings

FTIR spectra of neat and nanocomposite scaffolds are given in Fig. 4. The spectra of electrospun P(VDF-TrFE) indicates the presence of the α phase related bands at 509 and 764 cm^−1^ and the β phase related bands at 470, 845, 1080, 1180, 1284, 1400 cm^1^ **(**Fig. 4a**)** [35]. Intensity of IR bands corresponding to β phase was stronger than those corresponding to α phase in the case of all the samples (Fig. 4b). Figure 4b (Inset) shows the influence of TNW loading on the percentage of relative fraction of β-phase content (F(β)%) in P(VDF-TrFE). For neat electrospun P(VDF-TrFE), P(VDF-TrFE)/TNW-0.5 and P(VDF-TrFE)/TNW-1, F(β) were found to be about 76–77%. Whereas, for P(VDF-TrFE)/TNW-2, the F(β) was about 81%. In contrast, F(β) of P(VDF-TrFE)/TNW-3 was considerably less than the neat and composite scaffolds (about 66%). Thus, FTIR analysis demonstrated that the electroactive β phase crystallization was promoted by TNW at 2% w/w loading. However, higher content of TNW (3% w/w) inhibited the β phase formation.

#### XRD

**Fig. 5:**
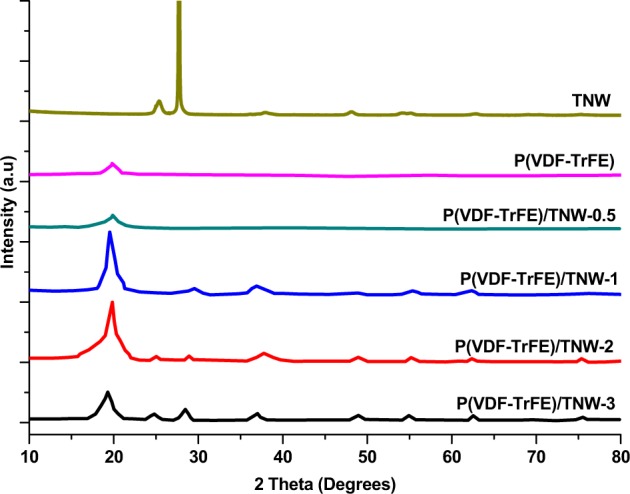
XRD patterns of the neat P(VDF-TrFE) and P(VDF-TrFE)/TNW nanocomposite membranes with increasing concentration of TNW

XRD analysis was conducted to determine the crystalline nature of P(VDF-TrFE) incorporated with TNW (Fig. 5). Neat P(VDF-TrFE) scaffolds showed characteristic diffraction pattern at 19.8° that indicating the α phase [36]. Peaks that correspond to the diffraction patterns of TNW were also observed in the XRD patterns of P(VDF-TrFE)/TNW composites scaffolds. The crystalline phase of P(VDF-TrFE) was not affected with the addition of small amount of TNW (≤2% w/w).

#### DSC analysis

**Fig. 6:**
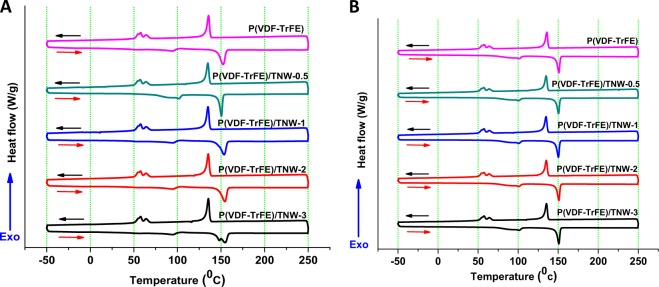
DSC thermograms showing first heating/cooling (**a**) and second heating/cooling (**b**) ramp of bare P(VDF-TrFE) and P(VDF-TrFE)/TNW nanocomposite scaffolds. Blue arrows on the Y-Axes indicate the direction of heat flow. Red (heating) and black (cooling) arrows inside the thermograms indicate the direction of heating/cooling

The thermal behavior and crystalline characteristics of P(VDF-TrFE) and its composites was evaluated using DSC analysis. The first (Fig. 6a) and second (Fig. 6b) heating and cooling cycles of the samples were recorded. Two endothermic peaks were observed at ~93 and 152.8 °C for P(VDF-TrFE) copolymer. Melting point (Tm) and crystallization point (Tc) were in the range of 150–153 and 134–136 °C respectively [37]. The thermal and crystallization characteristics of electrospun bare P(VDF-TrFE) and the composites were obtained from DSC analysis. The ferroelectric-paraelectric transition (T_F-P_) of P(VDF-TrFE) was observed around 93 °C and could be due to the possible melting of crystalline β-phases [38]. Another transition around 152.8 °C was observed as a result of the melting of all the crystalline phases (Tm) in the polymeric matrix. However, during the cooling step, bare scaffolds and nanocomposite scaffolds showed exothermic peaks of T_c_ and T_P-F_ around 134–135 and 57–59 °C, respectively. It was found that there was no considerable variation in any of the thermal transitions during the second heating and cooling cycle, in P(VDF-TrFE)/TNW scaffolds [39].

#### Tensile testing

**Fig. 7:**
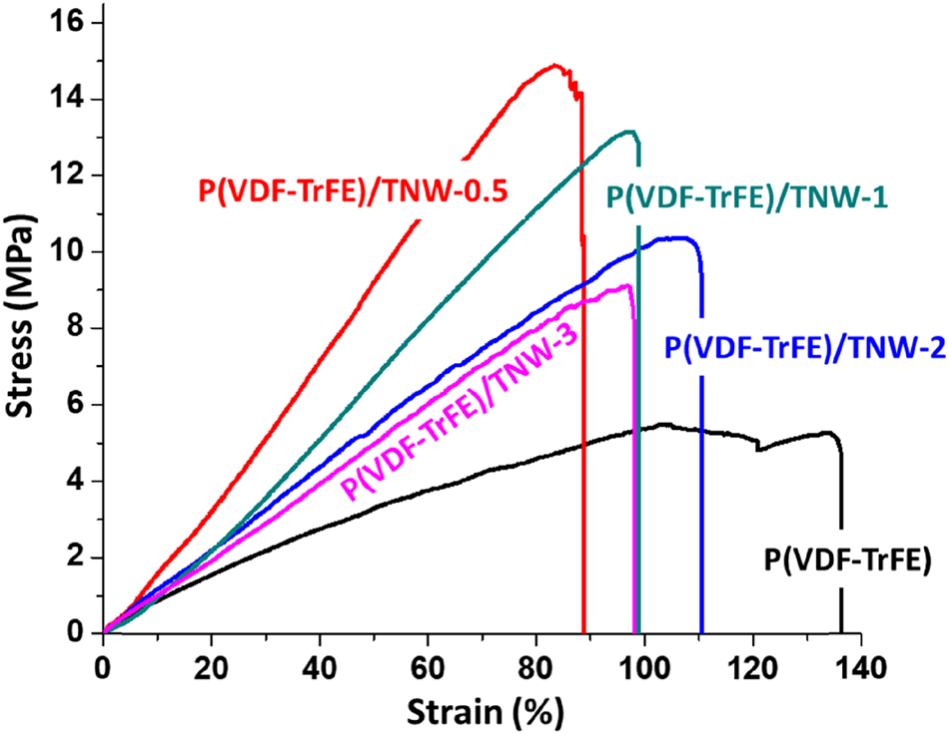
Tensile stress-strain curves of P(VDF-TrFE) and P(VDF-TrFE)/TNW nanocomposite scaffolds

The mechanical strength of the scaffolds with and without TNW was evaluated using tensile testing (Fig. 7 and Table 2). It was observed that the average elongation at break of pristine P(VDF-TrFE) scaffolds was 136.4 ± 4.2%. For the nanofibres that were incorporated with 0.5, 1, 2, and 3% w/w of TNW the elongation at break were 88.7 ± 6%, 98.7 ± 4.8%, 109.1 ± 5.2% and 97.5 ± 6.8% respectively. From this it was evident that the incorporation of TNW in the P(VDF-TrFE) nanofibres reduced the elasticity of P(VDF-TrFE). With the higher amount of TNW_,_ mechanical properties of the scaffold were considerable altered. Overall tensile strength of the scaffold increased with the introduction of certain quantity of TNW. For example, bare P(VDF-TrFE) showed an average tensile strength of 5.3 ± 2.3 MPa whereas P(VDF-TrFE)/TNW-0.5 showed an average tensile strength of 14.7 ± 3.8 MPa. A relatively similar result was obtained for P(VDF-TrFE)/TNW-1 (13.3 ± 1.8 MPa). However, a considerable reduction in the tensile strength was observed in the case of P(VDF-TrFE)/TNW-2 (10.3 ± 1.6 MPa) and P(VDF-TrFE)/TNW-3 (9.11 ± 2.3 MPa). Young’s modulus was increased from 8.3 ± 2.1 MPa to 17.8 ± 1.7 MPa upon the incorporation of 0.5 w/w TNW in P(VDF-TrFE) matrix. Higher TNW loading decreased the Young’s modulus of the nanocomposite scaffolds.

**Table 2:** Mechanical properties of electrospun P(VDF-TrFE)/TNW nanocomposite membrane

Samples	Maximum elongation (%)	Ultimate tensile strength (MPa)	Young’s modulus (MPa)
P(VDF-TrFE)	136.4 ± 4.2	5.3 ± 2.3	8.3 ± 2.1
P(VDF-TrFE)/TNW-0.5	88.7 ± 6.0	14.7 ± 3.8	17.8 ± 1.7
P(VDF-TrFE)/TNW-1	98.7 ± 4.8	13.3 ± 1.8	12.7 ± 1.5
P(VDF-TrFE)/TNW-2	110.9 ± 5.2	10.3 ± 1.6	10.4 ± 1.8
P(VDF-TrFE)/TNW-3	97.5 ± 6.8	9.11 ± 2.3	9.5 ± 2.3

#### Cell adhesion and cell viability of fibroblast and osteoblastic cells

**Fig. 8:**
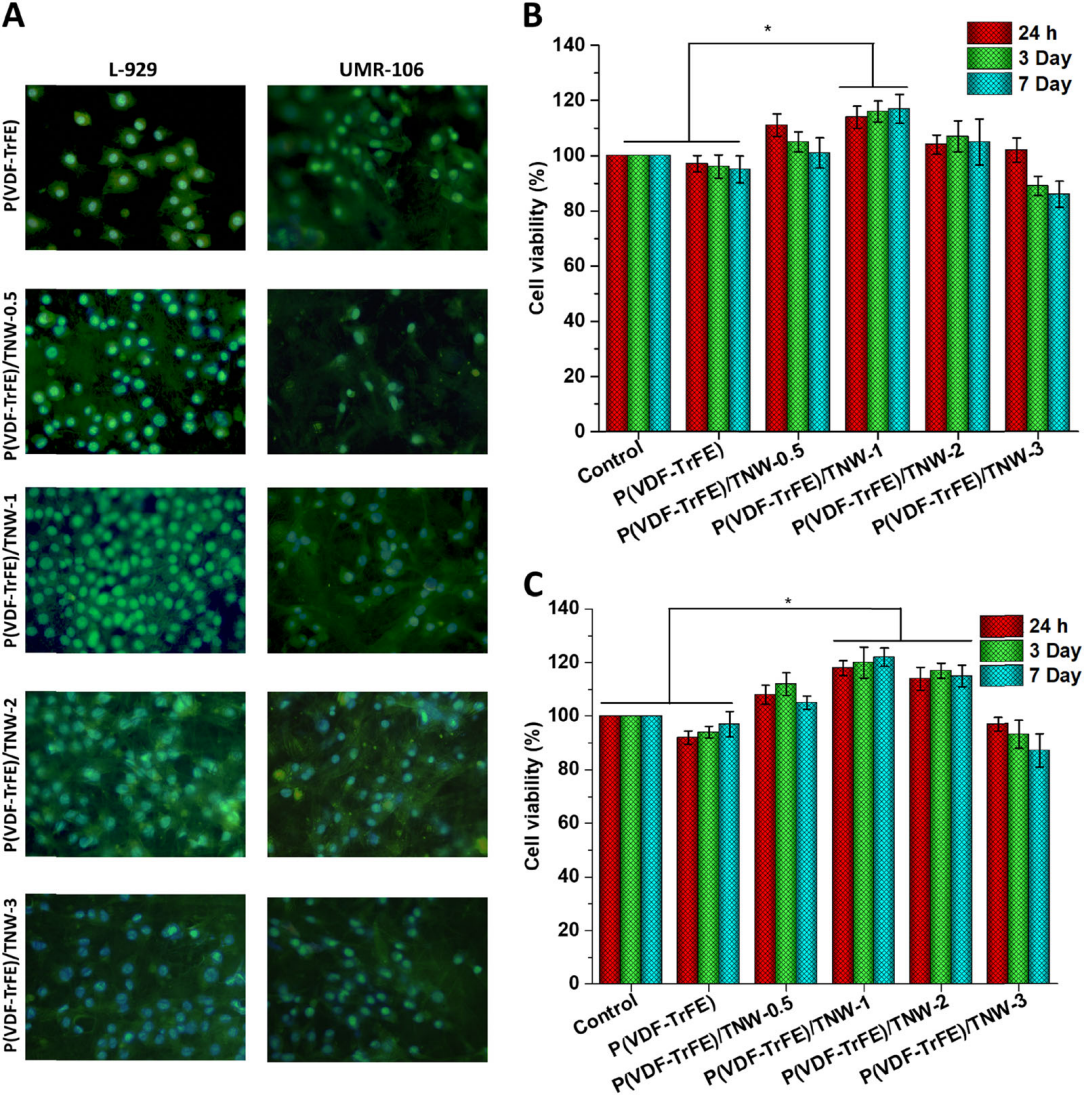
Adhesion of L-929 and UMR-106 cells to the scaffolds after 24 h of culture (**a**). Cell viability of L-929 cells (**b**) and UMR-106 cells (**c**) which were seeded on the scaffolds, as determined fby MTT assay. Data are the mean ± S.D. of three independent set of experiments

To verify the effect of fabricated P(VDF-TrFE)/TNW nanocomposite scaffolds in mammalian cell attachment, L-929 fibroblasts and UMR-106 osteoblast like cells were grown on the fabricated scaffolds and assessed the cell attachment and proliferation. Figure 8a. shows the microscopic images of the L-929 cell adhesion and proliferation on the scaffolds after 24 h of cell culture. Interestingly, P(VDF-TrFE)/TNW scaffolds displayed the proliferation of higher number of cells compared to the bare scaffolds. Considerably smaller number of cells were only detected on bare P(VDF-TrFE) and P(VDF-TrFE)/TNW-0.5 scaffolds. The highest cell adhesion was observed on P(VDF-TrFE)/TNW-1 scaffold. P(VDF-TrFE)/TNW-2 scaffolds also showed relatively higher cell proliferation. However, P(VDF-TrFE)/TNW-3 scaffolds exhibited the presence of relatively a smaller number of adhered cells than P(VDF-TrFE)/TNW-1.

To determine the viability of UMR-106 and L-929 cells proliferated on P(VDF-TrFE) and P(VDF-TrFE)/TNW scaffolds, MTT cell viability assay was carried out after 24 h, 3 days and 7 days and the results are given in Fig. 8b, c. Retention of **v**iability and functionality of the cells which are seeded on a the tissue engineered product is a key factor that determine the success of the engineered construct [40]. Obtained results clearly demonstrated that the presence of TNW in P(VDF-TrFE) does not considerably affect the cell viability upto 2% w/w loading. Cells seeded on P(VDF-TrFE)/TNW-1 and P(VDF-TrFE)/TNW-2 scaffolds exhibited highercell viability than the other samples studied (*P* ≤ 0.05).

## Discussions

Electrospun P(VDF-TrFE) based biomaterials are used as scaffolds for tissue engineering applications with promising results. However, additional approaches need to be undertaken to further improve the cell attachment and proliferation on these scaffolds. In this direction, we incorporated various amounts of titanium dioxide nanowires (TNW) in electrospun P(VDF-TrFE) polymeric scaffolds and assessed their applicability in bone tissue engineering by various physicochemical and biological tests.

TNW was synthesized by wet chemical approach. Calcination of synthesized amorphous TiO_2_ at high temperatures resulted in the formation of highly crystalline nanostructures [23, 41]. Calcination for 6 h at 500 °C converted the amorphous phase of TiO_2_ to crystalline anatase and rutile phases of TiO_2_ [41]. Observed broadening of major diffraction patterns might be due to the tiny size of the TiO_2_ nanocrystals [42]. TEM analysis indicated the formation of fiber like TiO_2_ structures which were further composed of smaller crystalline nanofibers. Synthesized TNW were incorporated in electrospun P(VDF-TrFE) scaffolds to improve the tensile strength and cell proliferation on the scaffolds. We observed a slight variation in the fiber diameter of the scaffolds containing TNW. Such a minor variation in fiber diameter can be due to the difference in solution properties such as viscosity and conductivity of the spinning solution when nanoparticles were incorporated in the polymer solution [43].

Results of FTIR analysis indicated that P(VDF-TrFE)/TNW-0.5 showed the presence of higher amount of piezo-active crystalline phase. This enhancement in the crystalline phase of P(VDF-TrFE) might be due to the nucleation effect of TNW. Small sized TiO_2_ nanoparticles (10 nm) can improve the crystallization of PVDF based polymers [44]. Addition of small quantity of nanofillers can improve the piezo-active phase in P(VDF-TrFE) [45, 46]. Relative increase in FTIR peak intensity of the peaks corresponding to β phase in the nanocomposite scaffolds (especially in P(VDF-TrFE)/TNW-2) could be due to the nucleating effect of TNW [38]. At higher TNW content, especially in P(VDF-TrFE)/TNW-3, the β phase peaks seem to be diminished. This can be supported by other studies where a reduction in the overall crystallinity of P(VDF-TrFE) was observed upon the incorporation of higher quantity of TiO_2_ nanomaterials [47]. The plausible reason for the observed decrease in the crystalline fraction at higher loading could be the formation of TNW agglomerates in the polymer matrix which might have affected β phase formation (by affecting nucleation). In addition, this can be due to the destruction of ordered arrangement of P(VDF-TrFE) copolymer due to the agglomerates and the subsequent decrease in crystallinity [48]. Based on the earlier studies, it can be believed that the decrease of crystallinity with increasing TNW content might be due to the interruption of crystal growth by some of the agglomerated TNW, however at low loadings, they acted as a nucleating agents [49]. This agrees with the results of XRD analysis where a considerable reduction in XRD peak intensity was observed in the case of P(VDF-TrFE)/TNW-3. In addition to the information regarding the crystallinity, XRD data confirmed the presence of TNW in P(VDF-TrFE) polymer matrix.

DSC analysis of the nanocomposite scaffolds was performed to understand the thermal transitions of P(VDF-TrFE) upon controlled heating and cooling. The ferroelectric-to-paraelectric transition (T_F-P_) of P(VDF-TrFE) was observed around 93 °C and could be due to the possible melting of crystalline β-phases [38]. Another transition near 152.76 °C was observed resulting from the possible melting of all the crystalline phases (Tm) in the P(VDF-TrFE). Above mentioned endothermic peak which was observed during melting was broad due to the merging of the melting peaks of the lower-melting α phase and the higher melting β phase [50]. During the first heating-cooling cycle of nanocomposite scaffolds, Tm and T_F-P_ showed a considerable alteration (Table 3). Highest variation was observed for P(VDF-TrFE)/TNW-0.5 scaffolds [51]. This might be due to the nucleation effect of TNW at lower concentrations (as explained in the previous section). However, during the cooling step, neat and nanocomposite scaffolds showed exothermic peaks of T_c_ and T_P-F_ at ~134–135 and 57–59 °C, respectively. There was no considerable variation in any of the thermal transitions during the second heating and cooling cycle, in P(VDF-TrFE)/TNW scaffolds [39]. This suggest that influence of TNW alone in the nucleation and crystallization of P(VDF-TrFE) was minimum [52]. However, the variation in thermal transitions during first heating/cooling step indicates that TNW played a prominent role in the crystallization during electrospinning process which may be also due to its effect on electrospinning parameters [53].

**Table 3:** Effect TNW on the thermal behavior of P(VDF-TrFE) scaffolds during the first heating and cooling ramp

Samples	T_m_ (°C)	T_F-P_ (°C)	T_c_ (°C)	T_P-F_ (°C)
P(VDF-TrFE)	152.8	93.0	135.9	58.0
P(VDF-TrFE)/TNW- 0.5	150.5	100.8	134.6	57.8
P(VDF-TrFE)/TNW-1	152.8	95.6	135.9	57.4
P(VDF-TrFE)/TNW-2	154.1	94.3	134.6	57.8
P(VDF-TrFE)/TNW-3	154.8	95.2	135.9	58.4

We performed uniaxial tensile testing of the scaffold to understand the effect of TNW in the tensile properties of the scaffolds. A very significant improvement in the tensile strength of the scaffolds was observed upon the addition of TNW. This might be due to the reinforcement effect of TNW at optimum loading [54]. Addition of higher quantity of TNW produced a considerable reduction of tensile strength of the nanocomposite scaffolds compared to those with low loadings. It was apparent that the addition of TNW beyond a critical value decreased the tensile strength due to the lowering of crystalline fraction in the polymer [55]. Furthermore, higher amount of TNW results in their agglomeration which affects their proper dispersion in the polymer matrix [56]. Agglomeration of the particles causes the imperfect distribution of the stress in the polymer matrix that results in the reduction of load bearing capacity of the scaffold. Moreover, fiber morphology of TNW also attribute an impact on the tensile properties of the nanocomposite. For instance, interactions at the interface of polymer and reinforcing agent will be higher for polymer nanocomposites with high aspect ratio nanofillers [57]. Relatively strong polymer-filler interactions at interfaces can result in higher degrees of stress transfer and, therefore, higher tensile strength and modulus [58]. Moreover, increased crystallinity of the polymer upon the addition of TNW might also have played a major role in the improvement in the mechanical strength. In light of the DSC results, nanocomposite scaffolds containing 0.5% w/w TNW showed the highest overall crystallinity (Table 3).

TNW loaded P(VDF-TrFE) scaffolds displayed the proliferation of higher number of cells compared to the neat scaffolds. This improvement might be due to the synergistic effect arising from the ability of TNW to improve cell proliferation and the beneficial effect of electrical signals generated by the piezoelectric scaffolds [59–61]. This can be further supported by the FTIR and XRD results where an enhancement of β crystalline phase was observed upon the addition of TNW. This increase in piezoelectric β crystalline fraction might be one of the reasons for the superior osteoblast and fibroblast cell adhesion and proliferation on P(VDF-TrFE)/TNW scaffolds [8]. Our results are supported by earlier report where piezoelectric PVDF produced higher bone formation compared to non-piezoelectric PVDF upon implantation in rats [62]. In addition, presence of TiO_2_ might have played an important role in cell proliferation due to the possible photocatalytic generation of superoxide (O_2_^•−^) and hydroxyl (OH^•^) radicals in an aqueous environment [63]. Controlled application of super oxides can play a vital role in the differentiation of osteoprogenitors [64] by acting at key steps in osteogenic gene regulation [65]. This is further supported by the results of our previous studies using tissue engineering scaffolds loaded with various metal oxide nanoparticles consistently demonstrated that metal oxide nanoparticles can enhance cell proliferation, angiogenesis and wound healing in vitro and in vivo conditions [8, 66–70]. Considering the growing attention of piezoelectric polymeric scaffolds especially in bone tissue engineering, P(VDF-TrFE)/TNW nanocomposites have a great potential for designing highly promising tissue engineered bone constructs. More detailed in vivo studies are required to understand the advantages and limitations of such scaffolds for extended period of implantation and clinical use.

## Conclusions

P(VDF-TrFE) scaffolds loaded with TNW (up to 3% w/w) were developed and characterized by various physical and biological tests to demonstrate their applicability in bone tissue engineering. From SEM, XRD and FTIR analysis, it was established that the TNW were successfully incorporated in the P(VDF-TrFE) fibers. Average diameter of P(VDF-TrFE) decreased with the increasing TNW concentration. Results of FTIR analysis indicated that the intensity of electroactive β phase increased due to the incorporation of TiO_2_ nanowires at optimum concentrations (about 2% w/w). X- ray diffraction studies showed that the crystallinity of P(VDF-TrFE)/TNW nanocomposite scaffolds improved at relatively small concentrations of TNW. A considerable enhancement in the mechanical strength of nanocomposite scaffolds was evident from the tensile testing results. Cell adhesion study using fibroblasts and osteoblasts showed that P(VDF-TrFE)/TNW scaffolds containing 1 to 2% w/w TNW promoted cell proliferation. Cell viability study confirmed cytocompatibility of the fabricated nanocomposite scaffolds. Overall, this study demonstrated that TNW at optimum concentrations can induce cell adhesion/proliferation in P(VDF-TrFE) tissue engineering scaffolds. Since osteoblast cells were able to adhere and grow well on the developed nanocomposite scaffolds, they can be used as potential candidates for bone tissue engineering applications.
